# Risk factors for pre-term birth in Iraq: a case-control study

**DOI:** 10.1186/1471-2393-6-13

**Published:** 2006-04-18

**Authors:** Samim A Al-Dabbagh, Wafa Y Al-Taee

**Affiliations:** 1 Department of Community Medicine, Mosul College of Medicine, Mosul, Iraq; 2 Nineveh Area Health Authority, Mosul, Iraq

## Abstract

**Background:**

Preterm birth (PTB)is a major clinical problem associated with perinatal mortality and morbidity. The aim of the present study is to identify risk factors associated with PTB in Mosul, Iraq.

**Methods:**

A case-control study was conducted in Mosul, Iraq, from 1^st ^September, 2003 to 28^th ^February, 2004.

**Results:**

A total of 200 cases of PTB and 200 controls of full-term births were screened and enrolled in the study. Forward logistic regression analysis was used in the analysis. Several significant risk associations between PTB and the following risk factors were identified: poor diet (OR = 4.33), heavy manual work (OR = 1.70), caring for domestic animals (OR = 5.06), urinary tract infection (OR = 2.85), anxiety (OR = 2.16), cervical incompetence (OR = 4.74), multiple pregnancies (OR = 7.51), direct trauma to abdomen (OR = 3.76) and abortion (OR = 6.36).

**Conclusion:**

The main determinants of PTB in Iraq were low socio-economic status and factors associated with it, such as heavy manual work and caring for domestic animals, in addition to urinary tract infections and poor obstetric history.

## Background

Pre-term birth (PTB) is a major determinant of neonatal mortality, morbidity and childhood disability and remains one of the most serious problems in obstetrics. PTB is defined as gestational age at birth of less than 37 completed gestational weeks. It is further classified into three main categories: mild, very pre-term and extremely pre-term for births occurring at 32–36 weeks, 28–31 weeks and less than 28 weeks respectively, with average frequencies of 85%, 10% and 5%, respectively [1-3]. Despite major preventive efforts, the incidence of PTB has remained constant at about 5–10% of live births in most countries over the past two decades [4-6].

In 75% of PTB cases no obvious causes have been established, but several etiological risk factors have been identified. Non-obstetric risk factors include: poor socio-economic status [7,8], maternal malnutrition [9,10], illiteracy [10,11], maternal age of <20 and >35 years [11,12], heavy manual work [13], cigarette smoking [14], long distance travel [15], and trauma [16]. Obstetric risk factors associated with PTB include: cervical incompetence, multiple gestations, short birth intervals, abortion, pre-labor premature rupture of membrane (PPROM) and previous PTB [9,12,13,15,17-19].

A number of other medical conditions have also been associated with PTB including: Diabetes mellitus, urinary and genital tract infections and psychological stress [[15,20], and [21]].

The aim of this study is to identify local risk factors that could be targeted to reduce the risk of PTB in Iraq.

## Methods

The study was conducted in Mosul, the second largest city in Iraq and the center of the Nineveh regional government district with a population of about 2,700,000. During the six-month period from 1 September 2003 to 28 February 2004 cases and controls were selected from the three main maternity hospitals in Mosul. To ensure that all available cases admitted to the three hospitals during the study period were enrolled, study staff visited the hospitals on a weekly schedule.

A case-control study design was used. Cases were defined as pregnant women with a live PTB (29–<37 weeks) by vaginal delivery or caesarean section. Controls were defined as pregnant women admitted to the same hospitals with a full-term live birth (≥37 weeks) by vaginal delivery or caesarean section. In general, pre-term cases were diagnosed in advance by the resident specialist. All eligible cases present during the study hospital visits were approached in the postpartum recovery room following the birth. A total of 217 cases were selected and 200 of them agreed to participate in the study. Each case was assigned a control, a woman in the nearest adjacent bed to the case participant who met the selection criteria. All subjects approached agreed to participate in the study.

All cases and controls were interviewed face-to-face using a specially designed questionnaire. In addition to general background information, respondents were asked questions about suspected risk factors. Socio-economic status was scored according to the presence or absence of a private car (scored as 1 or 0) and the number of electric appliances in the household (up to 2 = 1, 3 to 5 = 2, 6 or more = 3). Living quarters were categorized as adequate and inadequate, the latter consisting of tents, mud huts and/or in partially built houses with no water or electricity supply. An overall socio-economic status score was calculated for each subject; a score of 2 or less indicated low socioeconomic status and a score of 3 or more was defined as moderate to high socioeconomic status.

Information was also collected on the frequency of meat consumption; occasionally (once a week or less), frequently, or regularly (twice a week or more). Information about suspected risk factors for the present pregnancy and past obstetric history were obtained from the patient and/or from the clinical case record as applicable. The diagnosis of cervical incompetence was taken from clinical case notes (defined as dilatation of the cervix 2 cm or more in the first trimester by US examination).

The patient was asked about the diagnosis and treatment of four conditions during pregnancy: diabetes, typhoid fever, urinary tract infections and genital tract infections.

Finally, emotional disturbances occurring during pregnancy were assessed by two questions assessing severe fright and anxiety. Severe fright was defined as hearing unexpected bad news and/or experiencing military actions. Anxiety was assessed using a scale adopted by the American Psychiatric Association [23]. This includes a list of six questions regarding stressful life events including: restlessness, easy fatigability, muscular tension, sleep disturbances, irritability and difficulties concentrating. The presence of anxiety was established when the score was positive for ≥50% of the six questions.

Odds Ratios (OR) and 95% Confidence Intervals (CI) for the OR were calculated. The p value was based on the value of Z; a p value > 0.05 was not significant (NS). The dependent variable of the logistic regression was the presence or absence of PTB. A stepwise forward logistic regression was used. All variables were included in the initial analysis; the variable with the strongest association was estimated first, followed by all significant variables.

## Results

A total of 200 cases and 200 controls were included in the study. Table 1 shows the age distribution of the study population. A high-risk association was observed between PTB and pregnancy at young ages = 19 years, with an OR = 4.90.

**Table 1:** Distribution of study population by age.

**Age groups (years)**	**Cases (no.)**	**Controls (no.)**	**OR**	**95% CI**	**P value**
<19	41	10	4.90	2.38–10.09	<0.001
20–29	90	102	1.00	-	-
30–34	37	48	0.72	0.45–1.17	NS
35–39	18	30	0.56	0.30–1.04	NS
≥40	14	10	1.43	0.62–3.30	NS
all ages	200	200	-	-	-

Table 2 reveals the socio-economic background of the study population. Almost all study subjects were housewives. A significant risk association was present between PTB and occasional meat consumption (OR = 3.26), heavy manual work (OR = 2.80), low socio-economic status (OR = 2.60), illiteracy (OR = 2.67) and caring for domestic animals (OR = 4.93).

**Table 2:** Distribution of cases and controls according to socio-economic background

**Variables**	**Cases (no.)**	**Controls (no.)**	**OR**	**95% CI**	**P value**
**1. Frequency of meat consumption**					

occasional moderate-high	121 79	64 136	3.62	2.16–4.92	<0.001

**2. Manual work**					

heavy usual	140 60	91 109	2.80	1.86–4.23	<0.001

**3. Socio-economic status**					

low	165	129	2.60	1.63–4.15	<0.001
moderate-high	35	71			

**4. Occupation**					

housewife	198	196	2.02	0.37–11.16	N.S
worker	2	4			

**5. Level of education**					

illiterate	128	80	2.67	1.78–4.00	<0.001
6 years	65	103	0.45	0.30–0.68	<0.001
>6 years	7	17	1.00	-	-

**6. Presence of domestic animal**					

present	60	16	4.93	2.72–8.93	<0.001
absent	140	184			

**7. Housing**					

inadequate	17	10	1.77	0.79–3.97	NS
adequate	183	190			

The associations between PTB and other suspected risk factors are presented in Table 3. Among the risk variables of the current pregnancy and past obstetric history, those showing a significant risk association with PTB were cervical incompetence (OR = 3.11), multiple pregnancy (OR = 6.89), previous PTB (OR = 35.12), and direct trauma to the abdomen (OR = 3.86). Table 3 also illustrates a positive risk association between PTB and urinary tract infection (OR = 2.78), anxiety (OR = 1.80), more frequent antenatal visits due to pregnancy complications (OR = 2.04) and typhoid fever (OR = 2.40). The latter, however, does not reach statistical significance.

**Table 3:** Distribution of study population according to other suspected risk factors.

**Variables**	**Cases (no.)**	**Controls (no.)**	**OR**	**95% CI**	**P value**
**Parity**					
0–2	112	111	1.00	-	-
3	23	24	0.95	0.52–1.75	NS
4	22	24	0.91	0.49–1.68	NS
5+	43	41	1.06	0.65–1.72	NS
all categories	200	200	-	-	-

**Risk variables of present pregnancy**					

cervical incompetence	47	18	3.11	1.73–5.58	<0.001
multiple pregnancy	19	3	6.89	2.00–23.69	<0.001
accidental hemorrhage	9	4	2.31	0.70–7.64	NS
genital tract infection	48	45	1.09	0.69–1.74	NS

**Obstetric history**					

previous PTB	30	1	35.12	4.74–260.32	<0.001
abortion	3	1	3.03	0.31–29.38	NS
PPROM	14	10	1.43	0.62–3.30	NS

**Medical disease**					

typhoid fever	16	7	2.40	0.97–5.97	NS
diabetes	2	1	2.01	0.18–22.35	NS
urinary tract infection	82	40	2.78	1.78–4.35	<0.001

**Emotional disturbances**					

severe fright	67	63	1.10	0.72–1.67	NS
anxiety	87	60	1.80	1.19–2.72	<0. 01

**antenatal visits due to pregnancy complications**					

0	25	30	1.00	-	-
1–3	65	95	0.53	0.35–0.80	<0.001
≥4	110	75	2.04	1.37–3.04	<0.001

**cigarette smoking**					

smokers	4	5	0.80	0.21–3.02	NS

**long travel and trauma to abdomen**					

history of long travel	61	65	0.91	0.60–1.39	NS
trauma to abdomen	45	14	3.86	2.04–7.30	<0.001

Finally, a forward logistic regression model for the occurrence of PTB is presented in Table 4. Almost all risk factors found to be significantly associated with PTB remained significant in this analysis, except for age, socio-economic status, education and previous PTB.

**Table 4:** Forward logistic regression model for the occurrence of PTB.

**variables**	**OR**	**p-value**	**95% CI**	
			
			**lower**	**upper**
anxiety	2.16	<0.001	1.28	3.64
occasional meat consumption	4.33	<0.001	2.60	7.22
Presence of and caring for domestic animal	5.06	<0.001	2.44	10.58
manual work	1.70	<0.05	1.02	2.84
urinary tract infection	2.85	<0.001	1.63	4.98
direct trauma to the abdomen	3.76	<0.001	1.77	7.98
abortion	6.36	<0.001	2.77	14.63
cervical incompetence	4.74	<0.001	2.29	9.79
multiple gestation	7.51	<0.01	1.89	29.87

## Discussion

A case-control study design was conducted in which the response rate for the cases was 93% and for the controls 100%. Cases that declined to participate in the study cited fatigue as the reason. Recall bias is certainly one of the major limitations of a case-control study. This, however, is thought to be relatively moderate since the factors being assessed were related to pregnancy, which many women recall vividly. This assumption is reinforced by the fact that mothers were interviewed very soon after birth and after a thorough explanation of the study's aims. Efforts were also made to establish rapport between investigators and the study population.

PTB is one of the most common obstetric problems, and pre-term neonates are more likely to die than full-term infants. Furthermore, those who survive run a greater risk of disability [1,2]. In the crude analysis a significant risk association was found between PTB and women who conceived at younger but not at older ages. Age, however, became insignificant in the regression analysis when controlling for other variables. Contradicting results have been observed in other studies between the age of the mother at conception and PTB [11,23]. No significant association was observed between PTB and parity. Some cross-sectional analyses have reported an association with high parity, while others showed no effect of parity on the occurrence of PTB [24].

Frequency of meat consumption was used as an indicator of the woman's nutritional status and the study found that occasional as opposed to frequent meat consumption was significantly associated with PTB. Meat is expensive in Iraq and only higher-income families can afford frequent consumption. Meat is also considered to be an essential source of iron, and iron deficiency anemia has been regarded as a risk factor for PTB [25]. In Iraq such anemia is frequent among women and may be directly linked to lack of meat consumption [26]. Moreover high-risk pregnancies in Mosul have been significantly more prevalent among malnourished women [27].

Poor socio-economic background and illiteracy were also both found to be significantly associated with PTB. Similarly, significant associations were observed between PTB and heavy manual work and caring for domestic animals. All these conditions are interrelated and are proxies for low socio-economic status. This might explain why some of these factors became insignificant predictors of PTB in the forward logistic regression analysis. Similar results have been reported elsewhere. Other studies have also found that limiting the amount of work done by pregnant women and avoiding fatigue helps reduce the risk of PTB [8-10,13,15]. The study also revealed significant risk associations between the presence of cervical incompetence, multiple pregnancies and previous PTB. This, too, is in accordance with other studies [9,12,13,17]. Accidental hemorrhage has also been suspected as a risk factor [19]. In the present study, an OR of 2.31 for hemorrhage was found but was not significant. This might be due to the small number of cases detected.

Urinary tract infections were found to be a significant risk factor for PTB in this study, which reflects findings in some other studies [28]. No association, however, was observed between PTB and genital tract infection. Other studies are inconclusive. Although similarly negative associations have been reported, some other studies have found a positive association, particularly with trichomoniasis, bacterial vaginosis and mycoplasmal infections [21,29]. The failure in this study to find a positive association might be due to the study design. The incidence of these infections was determined by clinical case histories only and no direct laboratory results were available to the authors. It is possible that women may confuse the two infections or may be more prone to report urinary rather that genital infections.

The study also investigated the possible association of PTB with histories of other medical diseases. Only two cases of diabetes were observed among cases and one among controls. Typhoid fever (OR = 2.40) is relatively common in Mosul and patients usually correctly recall its history and treatment. The association between PTB and typhoid fever should be further investigated and documented by laboratory tests. Similar results elsewhere also associated maternal pyrexial illnesses with PTB [1].

Other studies have revealed controversial results for an association between smoking and PTB [30]. In this study too few smokers were observed to draw a valid conclusion. Due to social sitgma women in Iraq have been reluctant to state their smoking habits [31], so it is possible that the presence of smokers in this study has been undereported. With regard to long distance travel, our findings agree with Schoeman et al [32] in that it does not present a significant risk of PTB. Other studies, however, have found a significant association [15]. Direct trauma to the abdomen was reported by 45 cases and 14 controls and the association was significant, reflecting the findings of other studies [11,12].

Not surprisingly, the study found that cases had undergone a greater number of antenatal care visits than had the controls, mainly for pregnancy complications. The coverage of antenatal care is very low in Iraq, about 30%, and antenatal visits are mainly made for high-risk pregnancies[34]. The findings of this study support the view that women diagnosed with problems that may lead to PTB are more prone to use antenatal care services than others.

Stress, anxiety and other psychological disturbances have been suspected as risk factors for PTB [7,15,23]. It is a common belief in Iraqi communities that PTB is associated with anxiety and severe fright, and in the last two years stressful life events have increased in this country. The study confirmed a significant association between anxiety and PTB. It has been claimed that stress and anxiety increases corticotrophin-releasing hormones and may ultimately result in increased uterine contractility. Stress also increases cytokine production, which may independently lead to PTB or increase susceptibility to infection and subsequent PTB [35].

Many of the suspected risk factors listed above are interrelated with each other and probably with some other co-factors. Nevertheless, the majority of significant associations observed in the study remained so after conducting a forward logistic regression analysis.

## Conclusion

Mosul, in common with other parts of Iraq, has been affected by war and sanctions, and pregnant women are a particularly vulnerable group. They face the consequences of poor nutrition and even malnutrition, low socio-economic standards, infections and exposure to stress and anxiety. All these risk factors, which have been found to be associated with PTB, are modifiable. They should be taken into consideration in the planning of a preventive program to decrease PTB and its sequela for mortality and morbidity among infants in Iraq.

## Competing interests

The author(s) declared that they have no competing interests.

## Authors' contributions

Authors have equal contribution. Both authors read and approved the final manuscript.

## Pre-publication history

The pre-publication history for this paper can be accessed here:


